# A magnetoencephalography dataset for motor and cognitive imagery-based brain-computer interface

**DOI:** 10.1038/s41597-021-00899-7

**Published:** 2021-04-29

**Authors:** Dheeraj Rathee, Haider Raza, Sujit Roy, Girijesh Prasad

**Affiliations:** 1 School of Computer Science and Electronic Engineering, University of Essex, Colchester, CO4 3SQ United Kingdom; 2 School of Computing, Engineering & Intelligent System, Ulster University, Derry~Londonderry, BT48 7JL United Kingdom

**Keywords:** Brain imaging, Biomedical engineering

## Abstract

Recent advancements in magnetoencephalography (MEG)-based brain-computer interfaces (BCIs) have shown great potential. However, the performance of current MEG-BCI systems is still inadequate and one of the main reasons for this is the unavailability of open-source MEG-BCI datasets. MEG systems are expensive and hence MEG datasets are not readily available for researchers to develop effective and efficient BCI-related signal processing algorithms. In this work, we release a 306-channel MEG-BCI data recorded at 1KHz sampling frequency during four mental imagery tasks (i.e. hand imagery, feet imagery, subtraction imagery, and word generation imagery). The dataset contains two sessions of MEG recordings performed on separate days from 17 healthy participants using a typical BCI imagery paradigm. The current dataset will be the only publicly available MEG imagery BCI dataset as per our knowledge. The dataset can be used by the scientific community towards the development of novel pattern recognition machine learning methods to detect brain activities related to motor imagery and cognitive imagery tasks using MEG signals.

## Abstract (summary)

**Table:** 

Measurement(s)	brain physiology trait
Technology Type(s)	Magnetoencephalography
Factor Type(s)	age group • sex
Sample Characteristic - Organism	Homo sapiens

Machine-accessible metadata file describing the reported data: 10.6084/m9.figshare.13561976

## Background & Summary

Mental imagery activities such as imagination of limb movement or mathematical calculation induce explicit and predictive patterns of brain activity that can be detected using electroencephalography (EEG) or magnetoencephalography (MEG)^1^. One of the most prominent brain patterns is event-related desynchronization/synchronization (ERD/ERS) of brainwaves in the alpha (8–13 Hz) and beta (16–30 Hz) frequency bands during motor imagery tasks. Brain-computer interfaces (BCIs) can detect and translate these patterns into actions and thus, provide a potential medium for communication and rehabilitation for patients with severe neuromuscular impairment^2–4^. MI-based BCIs employed with neurofeedback training paradigms can induce brain plasticity and possibly contribute to the enhancement of motor rehabilitation for stoke patients^5–7^, thus, may provide an alternative to conventional recovery methods e.g. physical practice^8^ for these patients.

While majority of the research to date has focused on EEG modality, MEG can also be useful towards developing effective BCI systems^9,10^. MEG has the advantage of recording brain activity across the whole scalp while maintaining much higher spatial and temporal resolution. In addition, compared to EEG, MEG allows detection of higher frequencies as magnetic fields are less attenuated by the head bone and tissue as compared to electric fields^11^. Though not portable, MEG-based BCIs are relevant for rehabilitation interventions.

Regardless of great potential, MEG-based BCI systems still need significant improvement in terms of robust and efficient signal processing algorithms. A big constraint towards the development of novel algorithms or validating currently available BCI signal processing pipelines is lack of open source MEG-BCI datasets. As per our knowledge, there are no sizable datasets available currently. In this work, we publish an MEG-based BCI dataset recorded using a conventional BCI paradigm involving MI and cognitive imagery (CI) tasks. The dataset contains 1134 minutes of MEG recordings across 34 recording sessions of 17 healthy participants (two sessions for each participant recorded on different days), and 6,800 imagery trials. BCI interactions involved two MI (both hands and both feet imagination) and two CI (word generations and mathematical subtraction) states. On average, 66 minutes of MEG recordings and 400 imagery trials are available per participant. The dataset is one of the first MI- and CI-related MEG-based BCI datasets published to date and presents a significant step from existing datasets in terms of uniformity, state-of-the-art MEG system, number of participants and MEG channels.

## Methods

### Participants

The study involved recruitment of 20 healthy participants. However, data of three participants are excluded from the dataset due to quality issues. Thus, the current dataset consists of 17 participants including 14 males (82.35%) and 3 females (17.64%), wherein median age of participants is 28 years with minimum age 22 years and maximum age 40 years. Out of 17, 15 participants are right-handed and 2 participants are left-handed (by self reporting). Table 1 provides the demographic information of all the participants. The names of all participants have been hereby anonymised. The participants are identified only by their participant Ids i.e. *‘sub-1’* through *‘sub-20’*. Ids of excluded participants are *‘sub-5’*, *‘sub-8’* and *‘sub-10’*.

**Table 1:** Demographic information of all the participants with participant ID, age, gender, experience with BCI, and dominant hand.

Participant ID	Age	Gender	Exp with BCI	Dominant Hand
sub-1	37	M	Yes	L
sub-2	36	M	No	R
sub-3	23	M	No	R
sub-4	23	F	Yes	R
sub-6	32	F	No	R
sub-7	28	M	Yes	R
sub-9	32	M	No	R
sub-11	23	M	No	R
sub-12	29	M	Yes	R
sub-13	26	M	No	R
sub-14	30	F	No	L
sub-15	24	M	Yes	R
sub-16	36	M	No	R
sub-17	27	M	No	R
sub-18	40	M	No	R
sub-19	22	M	No	R
sub-20	23	M	No	R

The experimental procedures were approved by the University Research Ethics Committee of the Ulster University, Northern Ireland, UK. All research procedures were carried out in accordance with approved institutional guidelines and regulations and guidelines of the Helsinki declaration. Prior to the data acquisition process, all participants were informed about the purpose and the procedures of the experiments and informed consenting procedure was followed wherein participants provided written consent to allow usage of their anonymised data for research purposes by other researchers. The participants had been screened for the absence of any psychiatric condition, any medications taken, and contraindications to MEG. Inclusion criteria were as follows: healthy individuals, age between 18 to 80 years (both inclusive), and no history of neurological, developmental or language deficits. Exclusion criteria were as follows: claustrophobic, pregnant or breastfeeding, body tattoos, metal or active body implants and on-going medications.

### MEG data acquisition

MEG data were recorded with a 306-channel (102 magnetometers and 204 planar gradiometers) Elekta Neuromag^*TM*^ system (Elekta Oy, Helsinki, Finland) located at the Northern Ireland Functional Brain Mapping (NIFBM) Facility of the Intelligent Systems Research Centre, Ulster University. Elekta Neuromag^*TM*^ system (Elekta Oy, Helsinki, Finland) is installed with MaxShield^*TM*^ system which is a high-performance magnetic shielding system designed and optimised for bioelectromagnetic measurements using Elekta Neuromag^*TM*^. The system consists of structurally optimal magnetically shielded room with internal active shielding. All the participants were screened for any metallic foreign substance e.g. jewelry, coins, keys or any other ferromagnetic material before entering the magnetically shielded room. The standard fiducial landmarks (left and right pre – auricular points and Nasion), five head position indicator (HPI) coils (placed over scalp), and the additional reference points over the scalp were digitized (Fastrak Polhemus system) to store information about the participant’s head position, orientation, and shape. In addition, ocular and cardiac activities were recorded with two sets of bipolar electro – oculogram (EOG) electrodes (horizontal – EOG and vertical – EOG) and one set of electrocardiogram (EKG) electrodes, respectively. Before starting the data acquisition, the complete procedure and the experimental paradigm were described to the participants. All recordings were made with participants seated on a comfortable chair approximately 80 cm away from the projector screen and in upright position of MEG scanner. The MEG signals were filtered at a bandwidth of 0.01–300 Hz (online) and sampled at the rate of 1 kHz during the acquisition itself. Continuous head position estimation was started after 20 s of raw data recording and kept running for rest of the acquisition period.

### Experimental paradigm

**Fig. 1:**
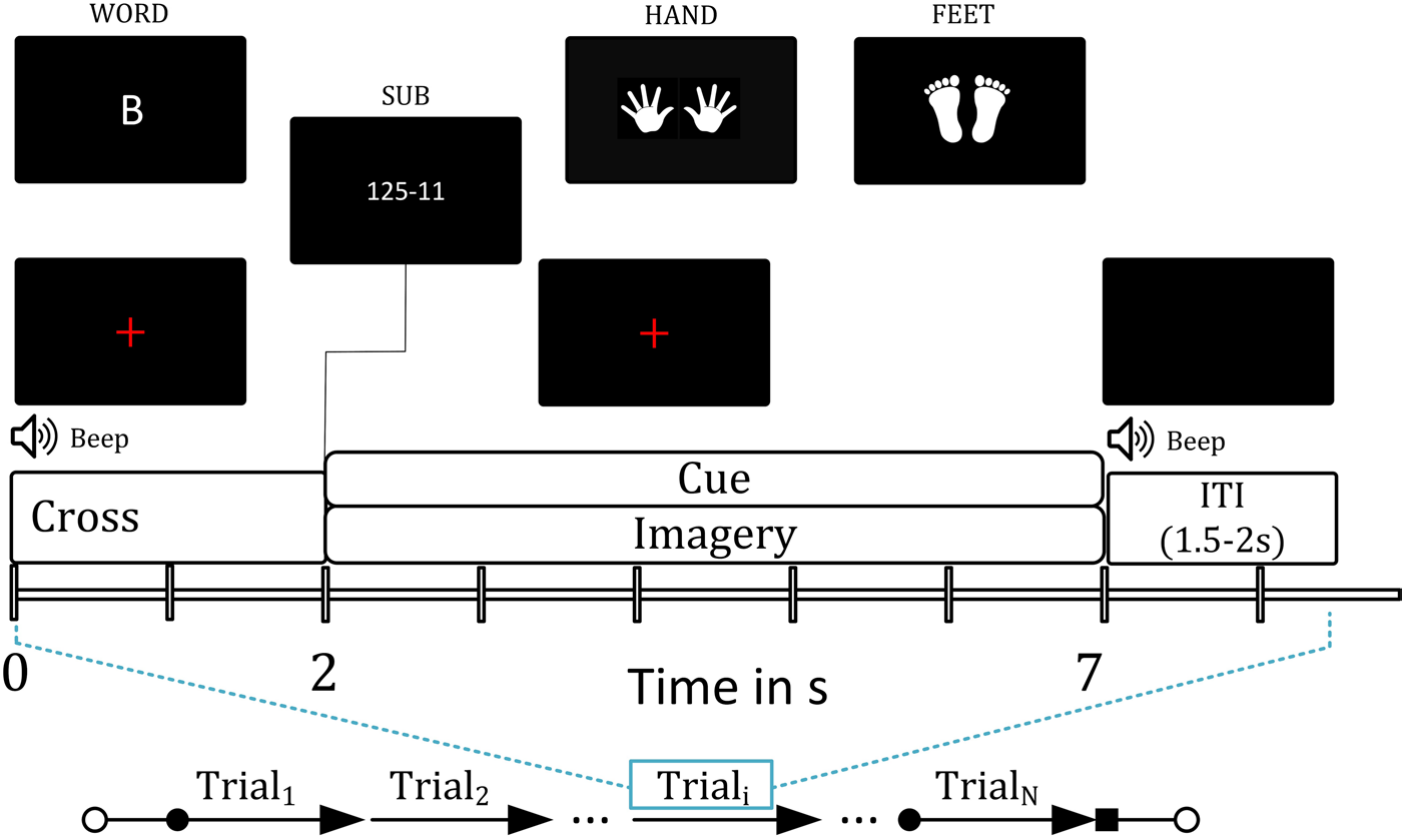
Timing diagram of MEG-BCI paradigm. Each trial starts with a rest period of 2 s followed by 5 s of imagery task period.

Figure 1 presents the timing diagram of the BCI paradigm used for the data acquisition. Each trial starts with a rest period of 2 s followed by 5 s of imagery task period. The cue remains visible during the whole imagery task period. During the rest period, participants were asked to fixate on a red cross presented at the center of the screen. A randomly selected inter- trial – interval (ITI) of 1.5–2 s was presented after the imagery task period. The fixation point and the cue were displayed on a Panasonic projector with a screen resolution of 1024 × 768 and refresh rate of 60 Hz. MEG data were acquired over 2 sessions (each session on different days) using the same BCI paradigm. Each session consisted of 50 trials for each of the imagery tasks, thus includes a total of 200 trials. A break of 5 minute duration was provided in each session after completion of first 100 trials. The participants were kept seated during the break and asked to relax.

The experimental paradigm was designed to cover four mental imagery tasks: two related to MI i.e. both hands movement imagery, both feet movement imagery and two related to CI i.e. mathematical subtraction imagery and word generation imagery. During the MI-related tasks, participants imagined movement of both hands/both feet when the related cue appeared at the screen (i.e. during the task period). Similarly, for CI tasks, participants either subtracted two numbers presented as cue or generated words related to the English language alphabet appeared as cue. Triggers were recorded within the *.fif* files (Elekta Neuromag^*TM*^ system) to mark the start of imagery period for each trial.

### Data processing

The original MEG dataset was acquired from all 306 sensors (204 gradiometers and 102 magnetometers) during two different sessions for each participant and recorded as *.fif* files. As each session consists of two data files due to session break, for better handling of the data, we have merged these files to create one single *.fif* file for each session. Thus, there are two raw *.fif* data files for each participant (i.e. one for each session). Our aim here is to provide the BCI researchers least processed data to allow them with greater flexibility towards customising the processing pipeline. However, we have also processed the *.fif* file format to convert the data in an epoched format (*.mat* file) to be compatible for BCI related analysis. Each epoch (trial) is generated with time duration of 7000 ms i.e. 2000 ms (pre-stimulus) to 5000 ms (post-stimulus). The triggers are available in both BIDS and.mat formats, where the classes are defined as follows:- Class 1: Both Hand Imagery, Class 2: Both Feet Imagery, Class 3: Word generation Imagery, and Class 4: Subtraction Imagery and their associated triggers in the STIM channels are 4, 8, 16, and 32, respectively. A detailed description of the data file structure is presented in Section ‘Data Records’. The fieldTrip Toolbox^12^ has been used in all data processing steps.

## Data Records

**Fig. 2:**
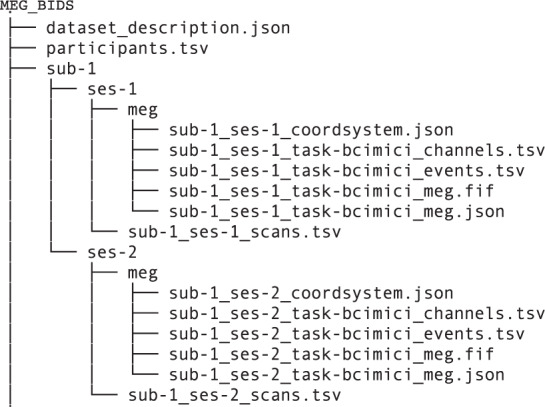
The structure of BIDS format data directory, where *MEG_BIDS* is a root folder. Under *MEG_BIDS* folder, each participant has its data folder (e.g. *sub*–1 is for participant 1), where two sub-folders are given for Session 1 and Session 2 of data recording, each sub-folder has a *meg* folder, where all the required information is available and *‘.fif’* files contain the MEG recording.

The data acquired during the described experiment are freely accessible and may be downloaded from figshare^13^, which is a general-purpose repository that makes research outputs available in a citable, shareable, and discover-able manner. It is worth to be noted that the data is available in two data formats i.e. MEG-BIDS format^14^ (*.fif*) and MATLAB compatible (*.mat*) file at the repository. Figure 2 shows the structure of the data directory for MEG-BIDS format where only one participant data structure is illustrated to avoid repetition. The folder named ‘MEG_BIDS’ contain two files named ‘dataset_description.json’ and ‘participant.tsv’. Further, there are 17 sub-folders (one for each participant data), each having scan file ‘_scan.tsv’ and a sub-folder named ‘meg’. Each ‘meg’ folder contains five files i.e. ‘_coordsystem.json’, ‘_channels.tsv’, ‘_events.tsv’, ‘_meg.fif’, and ‘_meg.json’.

We have also provided data in Matlab compatible format and shared the script at GitHub as well to convert the MEG-BIDS format to *.mat* file format. The root database folder (*MEG_mat*) contains two folders, namely *Session_01* and *Session_02*, which store datasets recorded on day 1 and day 2, respectively. Within each session folder, there are seventeen*.mat* files i.e. one for each participant. We have used a similar name convention for all files within the database e.g. in *sub-1_ses-1_task-bcimici_meg.mat* filename, *‘sub-1’* shows participant Id and *‘ses-1’* stands for session number. Each of these *.mat* files contains a Matlab structure with name *‘dataMAT’*. Table 2 provides names, data type, data size, and description of all the fields present within the *‘dataMAT’* Matlab structure. To provide more flexibility to readers, we have provided the data in both *BIDS and .mat* file format, which can be downloaded from figshare^13^. The root database folder is (*MEG_BIDS*) for BIDS format and (*MEG_mat*) for Matlab.

**Table 2:** Description of the fields present in the ‘.mat’ files for *MEG_mat* folder.

Field Name	Type & Size	Description
label	cell array [306 × 1]	MEG Channel labels
time	cell array [1 × 200]	Time stamps in accordance with cue
trail	cell array [1 × 200]	MEG data for 200 trials
fsample	array [1]	Sampling frequency
trialinfo	array [200 × 1]	Class labels of 200 trials
grad	structure [1 × 1]	A structure containing detailed information about the MEG sensors
trialclass	cell array [4 × 2]	Classes in number and string information about the MEG sensors

Each session has 200 trials, stored in a cell array [1 × 200], named *‘data.trial’*, and each trial has data from 306 channels for 7 s time duration (i.e. [306 × 7000]), where sampling frequency is 1000 Hz. The class labels are stored in *‘data.trialinfo’* which is an array of size [200 × 1].

## Technical Validation

We performed a technical validation of the dataset by estimating and evaluating spatio-temporal features for six binary-classification tasks. For this analysis, MEG data from 204 gradiometer sensors were used while discarding the data from 102 magnetometers, as former provide higher sensor-to-noise ratio and are more sensitive to cortical activations. It is well known that SMRs are more prominent in cortical brain regions. Further, we have selected data for a 3 s time duration i.e. from 0.5 s to 3.5 s after the onset of imagery task. To generate spatio-temporal features, one of the state-of-the-art methods (i.e. filter-bank common spatial pattern (FBCSP)) was employed. This method involves two main steps i.e. band-pass filtering within different frequency ranges (creating a filter-bank) and estimation of CSP features using the band-pass filtered data from previous step^15^. To explore the effect of selecting different combinations of frequency ranges, two filter-banks, namely FB1 and FB2, were created and CSP features were generated for both filter-banks separately. FB1 consisted of two frequency ranges i.e. 8–12 Hz – alpha (*α*) band and 14–30 Hz– beta (*β*) band. FB2 consisted of ten overlapping frequency ranges i.e. 8–12 Hz, 10–14 Hz, 12–16 Hz, 14–18 Hz, 16–20 Hz, 18–22 Hz, 20–24 Hz, 22–26 Hz, 24–28 Hz, and 26–30 Hz.

To evaluate the BCI performance, classification accuracies (CAs) were estimated by using a support vector machine (SVM) classifier for six binary classification tasks, i.e. hand versus feet (H-F), hand versus word generation (H-W), hand versus subtraction (H-S), feet versus word generation (F-W), feet versus subtraction (F-S), and word generation versus subtraction (W-S). This evaluation was performed for both intra-session condition (i.e. 10-fold cross-validation using Session 1 data) and inter-session condition (i.e. training of classifier with feature set of Session 1 data and evaluation on feature set of Session 2 data). The main reason for using 10-fold cross-validation estimator is that is has a lower variance than a single hold-out set estimator, which can be important if the amount of available data is limited as in our case we have 200 trials in each session.

**Fig. 3:**
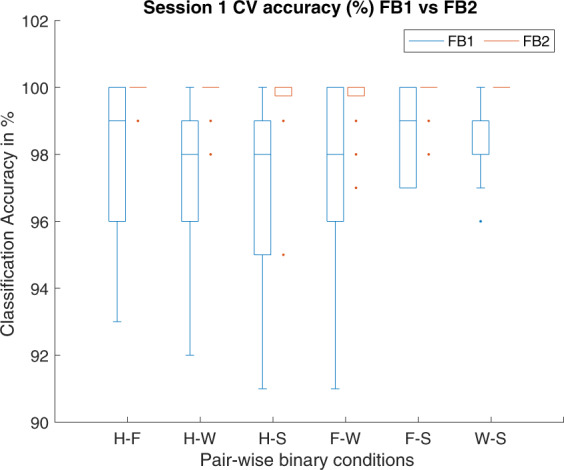
10-fold cross-validation accuracy for Session 1 data under two different filter-banks (1) FB1: Alpha-Beta; (2) FB2: 8–30 Hz, for 6 different binary task comparison (i.e. H-F, H–W, H–S, F–W, F–S, and W-S).

**Fig. 4:**
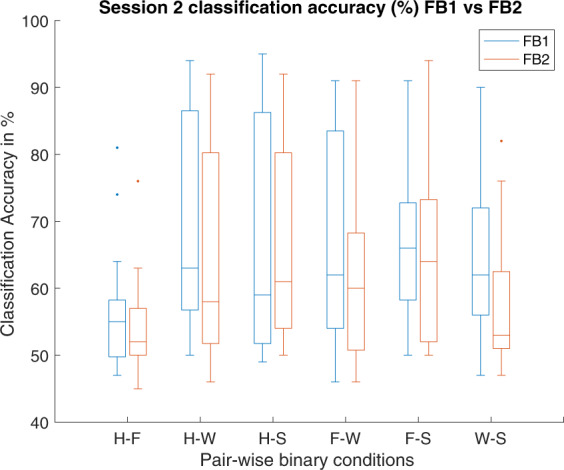
Inter-session classification accuracy-based performance comparison under two conditions FB1 and FB2 for six binary classification tasks.

The 10-fold cross-validation (intra-session condition) performance is reported using box plot in Fig. 3 with both filter-bank combinations (i.e. FB1 and FB2) for 6 different binary tasks comparisons (i.e. H-F, H-W, H-S, F-W, F-S, and W-S). The CA for FB1 ranged from 96.29% to 98.29% and for FB2 range from 99% to 99.94%. The overall results showed a high separability between the feature sets of different classes. The results for inter-session condition are reported in Tables 3 and 4 for FB1 and FB2, respectively. For FB1 which includes *α* and *β* frequency bands, H-W (i.e. hand vs word) class pair has achieved maximum average (over 17 participants) classification accuracy (i.e. 69.35%), wherein participant sub-3 performed best with 94% and sub-4 has the lowest accuracy of 50%. In FB2, H-S (i.e. hand vs subtraction) class pair has achieved maximum average classification accuracy (i.e. 66.65%), wherein participant sub-20 performed best with 93% and sub-4 has the lowest accuracy 50%. Figure 4 shows comparison between average classification accuracies of FB1 and FB2 for six binary classification tasks in inter-session condition. Here, FB1 performed better than FB2 for majority of class pairs.

**Table 3:** Inter-session single-trial classification accuracy (%) for condition FB1 i.e.

Participant ID	H-F	H-W	H-S	F-W	F-S	W-S
sub-1	58	53	53	61	51	51
sub-2	57	74	60	85	67	72
sub-3	74	94	95	70	69	74
sub-4	50	50	50	50	50	49
sub-6	47	54	50	58	57	53
sub-7	51	69	62	87	66	58
sub-9	51	67	83	50	75	70
sub-11	47	86	91	83	85	90
sub-12	56	56	51	68	59	63
sub-13	49	90	86	91	87	57
sub-14	50	62	50	54	55	65
sub-15	80	65	56	69	75	59
sub-16	57	62	57	62	70	72
sub-17	55	57	71	55	70	47
sub-18	53	88	88	45	64	60
sub-19	54	61	56	57	63	62
sub-20	64	91	90	87	91	77
Mean	56.06	69.35	67.59	66.59	67.88	63.47
std	9.07	14.92	17.12	4.97	12.03	11.29

8–12 Hz (*α*) and 14–30 Hz *β* frequency bands. H: Hand; F: Feet; W: Word; and S: subtraction.

**Table 4:** Inter-session single-trial classification accuracy (%) for condition FB2 i.e.

Participant ID	H-F	H-W	H-S	F-W	F-S	W-S
sub-1	59	44	53	56	50	50
sub-2	51	58	61	75	65	54
sub-3	76	92	89	64	52	72
sub-4	50	52	50	50	46	50
sub-6	55	52	71	50	63	52
sub-7	52	50	57	50	53	50
sub-9	51	52	50	50	50	51
sub-11	49	60	90	55	74	52
sub-12	55	50	50	65	52	64
sub-13	47	88	71	91	87	54
sub-14	50	67	55	52	55	76
sub-15	66	86	78	77	82	56
sub-16	58	69	62	63	74	67
sub-17	49	61	62	59	65	57
sub-18	50	79	87	44	65	46
sub-19	50	58	55	64	63	51
sub-20	64	93	92	87	93	84
Mean	54.82	65.35	66.65	61.88	64.06	58.00
std	7.68	16.27	15.26	15.26	13.63	10.77

ten overlapping frequency bands in range between 8–30 Hz. H: Hand; F: Feet; W: Word; and S: subtraction.

**Fig. 5:**
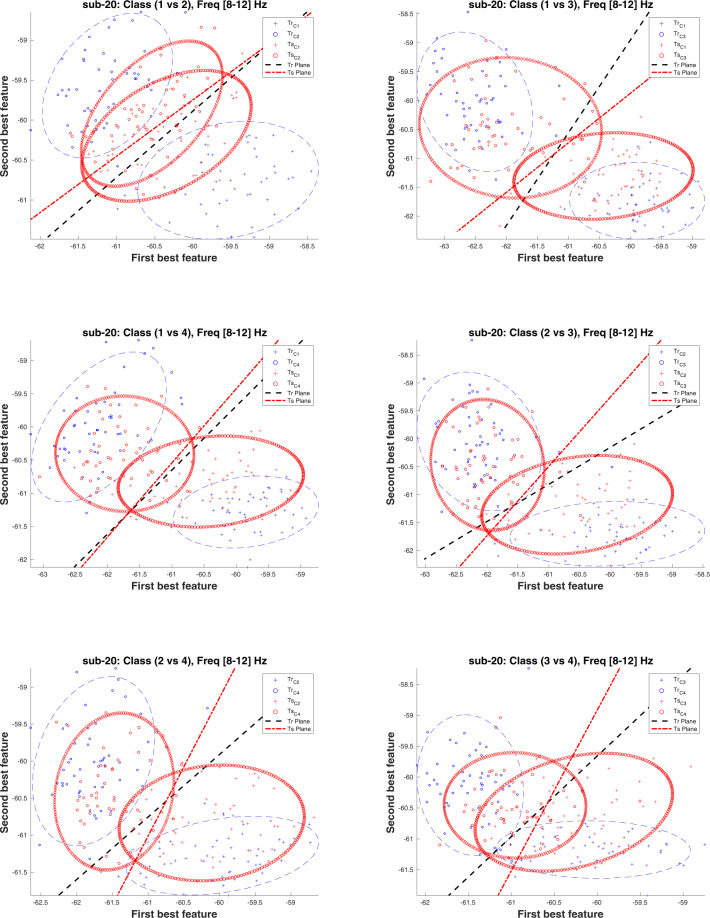
Covariate shift (CS) between the training (Tr) (i.e. Session 1) and test (Ts) (i.e. Session 2) distributions in the *α* band (i.e. 8–12 Hz) of participant sub-20 dataset for different binary class combinations, where Class 1: Hand, Class 2: Feet, Class 3: Word, and Class 4: Subtraction.

**Fig. 6:**
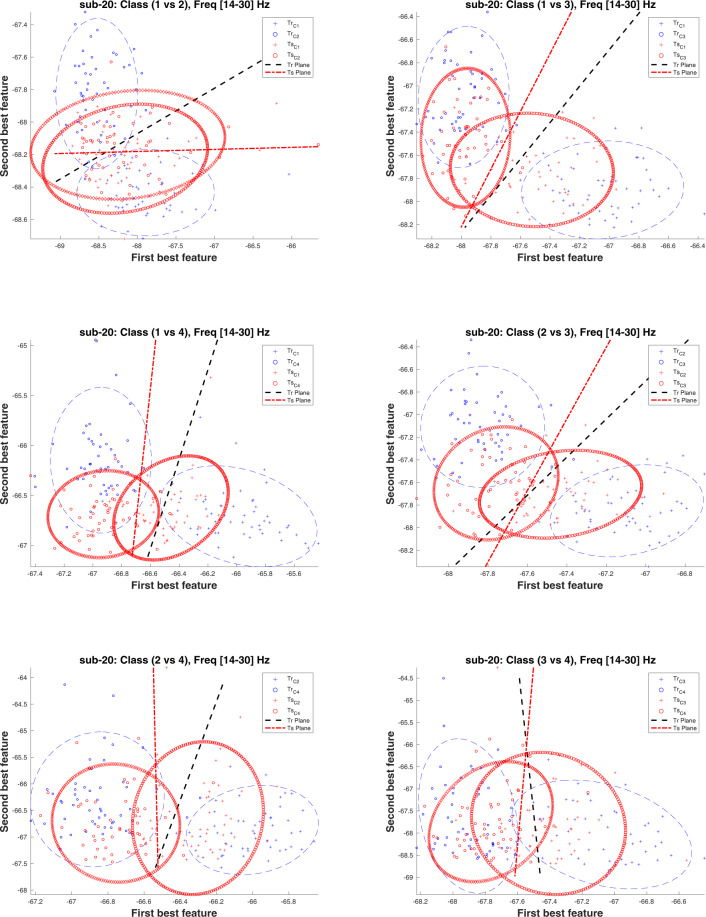
Covariate shift (CS) between the training (Tr) (i.e. Session 1) and test (Ts) (i.e. Session 2) distributions in the *β* band (i.e. 14–30 Hz) of participant sub-20 dataset for different binary class combinations, where Class 1: Hand, Class 2: Feet, Class 3: Word, and Class 4: Subtraction.

Notably, the CAs for inter-session condition is significantly lower than the intra-session condition for all binary classification tasks. Importantly, most of the machine learning methods in BCI are facing an issue of low performance in terms of classification accuracy, which may be due to the presence of non-stationarity in the data recorded over multiple sessions^16,17^. According to the literature, there are several reasons for the presence of non-stationarity in the data such as head movement, user fatigue, change in mood, or external noise interfering the MEG system^18^. We believe that the low CAs in the inter-session condition may be due to the presence of high non-stationarity (i.e. covariate shift) between MEG data of Session 1 and Session 2. The covariate shift is a case, where the input distribution of the data shifts (i.e. (*P*_*train*_(*x*) ≠ *P*_*test*_(*x*))), whereas the conditional probability remains the same, while transitioning from the training to testing stage, which in our case is from Session 1 to Session 2^19–21^). The covariate shift between Session 1 and Session 2 is a challenging issue, as demonstrated by a large difference between the performances of single-trial classification, wherein 10-fold cross-validation average accuracy on Session 1 data is significantly higher than evaluation average accuracy on Session 2 data. We have examined input data distribution between Session 1 and Session 2 for all participants and found that all the participants’ data had some form of covariate shift. Figures 5 and 6 illustrate the presence of covariate shift in the feature set of the participant *sub-20* for of *α* and *β* frequency bands, respectively. It is to be noted that the sub-20 data provided highest inter-session classification accuracy. Each figure consists of six sub-figures representing distribution between class pairs of six binary classification tasks. In each sub-figure, two ellipses with blue dashed line show the training distribution (*P*_*train*_(*x*)) for the two participating classes (e.g. two classes for top-left sub-figure in Fig. 5 are Hand and Foot imagery) and black dashed line presents the decision hyper-plane for the training dataset. Similarly, the ellipses with red points boundary show the test data distribution *P*_*test*_(*x*) for the same classes and the red dash line presents the decision hyper-plane for the test dataset. A clear shift pattern for the datasets can be seen within both Figs. 5 and 6, i.e. for majority of the class pairs, the training data has high separability as compared to the test data and there are large shifts in decision hyper-planes in most cases. This variation in inter-class separability may explain the low classification accuracies while evaluating the trained classifier with Session 2 data.

## Usage Notes

There are several potential uses for this database. Firstly, it can be used to test the effectiveness of already developed EEG-BCI data analysis pipelines using this MEG dataset. Secondly, we encourage any use that can contribute towards development of novel pattern recognition and machine learning methods to detect brain activities related to MI and CI tasks using MEG signals. Thirdly, since we have performed a basic analysis and single-trial classification of tasks using the raw data, future work may involve exploring impact of various MEG pre-processing methods e.g. head movement correction and maxfiltering^22^. Additionally, as the dataset contains two sessions that were recorded on different days for each participant, robustness of analysis pipelines towards inter-session non-stationarity can be assessed using this dataset. More importantly very high spatial resolution of MEG facilitates much enhanced source-level analysis. The data-sets can used for investigating source level features in accounting for inherent non-stationarity present in MEG neuro-imaging modality primarily due to head movements.

## Data Availability

The pre-processing and feature extraction of the MEG data, as well as the single-trial classification were performed using custom Matlab codes based on Fieldtrip toolbox^12^ functions. All codes are available at our GitHub repository https://github.com/sagihaider/MEGBCI2020.git.
